# Crystal structure of 8-(4-methyl­phen­yl)-2′-de­oxy­adenosine hemihydrate

**DOI:** 10.1107/S2056989017017212

**Published:** 2018-01-01

**Authors:** Ajaykumar V. Ardhapure, Yogesh S. Sanghvi, Yulia Borozdina, Anant Ramakant Kapdi, Carola Schulzke

**Affiliations:** a Department of Chemistry, Institute of Chemical Technology, Nathalal Parekh Road, Matunga, Mumbai 400 019, India; b Rasayan Inc. 2802, Crystal Ridge Road, Encinitas, California 92024-6615, USA; c Max Planck Institute for Biological Cybernetics Spemannstrasse 41, D-72076 Tübingen, Germany; d Institut für Biochemie, Ernst-Moritz-Arndt Universität Greifswald, Felix-Hausdorff-Strasse 4, D-17487 Greifswald, Germany

**Keywords:** crystal structure, adenosine, nucleoside, palladium, catalysis, Suzuki–Miyaura cross-coupling

## Abstract

In the asymmetric unit, equalling the unit cell (triclinic, *P*1, *Z* = 1), two mol­ecules of the title compound, 8-(4-methyl­phen­yl)-d-2′-de­oxy­adenosine, C_17_H_19_N_5_O_3_, are present, with distinct conformations of the two sugar moieties, together with one solvent water mol­ecule. All three ribose O atoms are involved in hydrogen bonding and the crystal packing is largely determined by hydrogen-bonding or hydrogen–heteroatom inter­actions (O—H⋯O, O—H⋯N, N—H⋯O, C—H⋯O and C—H⋯N) with one independent mol­ecule directly linked to four neighbouring mol­ecules and the other mol­ecule directly linked to six neighbouring mol­ecules. The two independent mol­ecules of the asymmetric unit display three weak intra­molecular C—H-to-heteroatom contacts, two of which are very similar despite the different conformations of the deoxyribosyl moieties. The aromatic ring systems of both mol­ecules are in proximity to each other and somehow aligned, though not coplanar. The absolute structures of the two mol­ecules were assumed with reference to the reactant 8-bromo-d-2′-de­oxy­adenosine as they could not be determined crystallographically.

## Abstract 2

8-(4-Methyl­phen­yl)-2′-de­oxy­adenosine was synthesized using a Suzuki–Miyaura cross-coupling reaction of 8-bromo-d-2′-de­oxy­adenosine and 4-methyl­phenyl­boronic acid in the presence of Pd(OAc)_2_ and a salton-derived ligand as a highly catalytically active system. There are two independent mol­ecules plus one solvent water in the asymmetric unit and the packing in the crystal lattice is heavily influenced by hydrogen bonding

## Chemical context   

Alkyl, alkenyl or alkynyl modified purines are known for having inter­esting biological activities. Many of these modified nucleosides show, for instance, potential for/activity as drug candidates, biological probes *etc* (Manfredini *et al.*, 1995 ▸). Attempts to implement green, *i.e*. eco-friendly, procedures for the synthesis of modified nucleosides involve the use of palladium complexes as active catalysts because of their proven ability to perform such catalytic transformations even in aqueous media (Agrofoglio *et al.*, 2003 ▸; Gayakhe *et al.*, 2016 ▸). Modifying the nucleoside bases by substitution of the C—H functions of purine and pyrimidine can be utilized for instance to install or increase fluorescence properties. Fluorescent nucleosides might then be employed as probes for studying the impact of changes in the biological environment: DNA damage, drug–DNA or protein–DNA inter­actions for instance. Such DNA probes are relevant for both chemical biologists as well as bio-organic chemists (Tanpure *et al.*, 2013 ▸). Structural elucidation of substituted nucleosides in general will aid our understanding of mechanistic aspects in this respect and provide a basis for *in silico* studies. The synthesis and crystal structure of the title compound, 8-(4-methyl­phen­yl)-d-2′-de­oxy­adenosine are presented here as part of our studies in this regard.
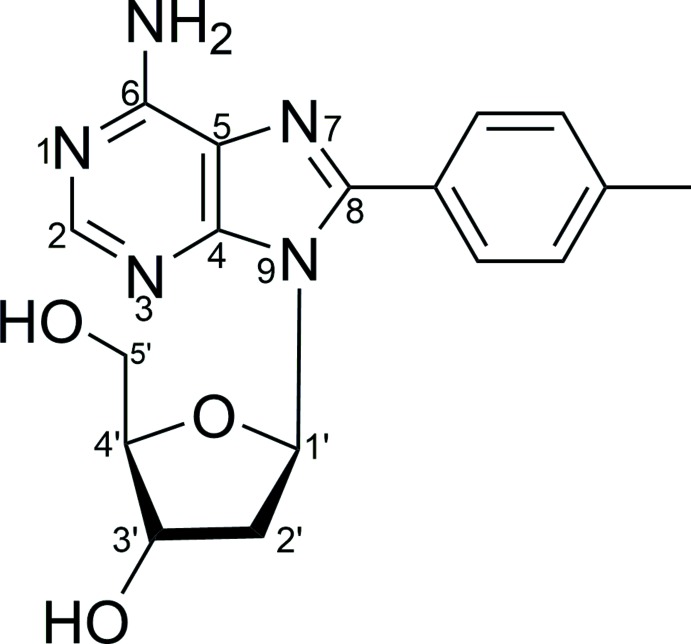


## Structural commentary   

**Figure 1:**
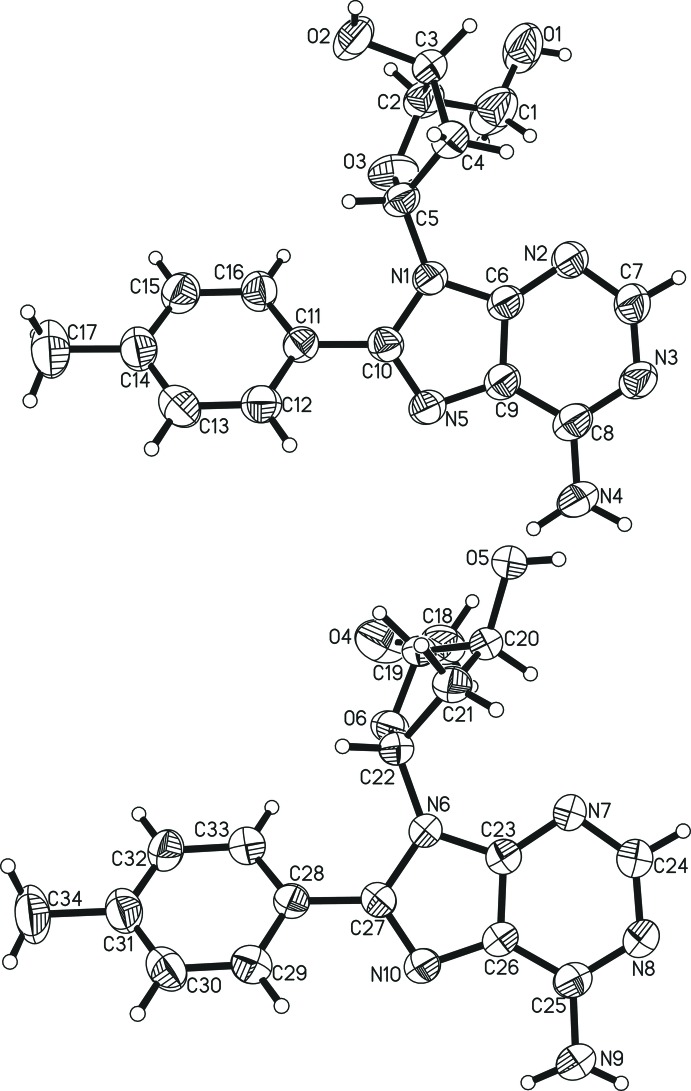
The two independent compound mol­ecules of the asymmetric unit displayed in a comparable orientation to show the distinct conformation of the deoxyribosyl moiety (top: mole 1; bottom: mole 2). Displacement ellipsoids are shown at the 50% probability level.

In the title compound, two mol­ecules of C_17_H_19_N_5_O_3_ crystallize together with one mol­ecule of water in the triclinic space group *P*1 with *Z* = 1. The two mol­ecules (mole 1 and mole 2, Figs. 1 ▸ and 2 ▸) differ in the puckering of the deoxyribose sugar, which is the most inter­esting feature of this novel mol­ecular structure. In mole 1 with 3′-exo puckering, the –CH_2_—OH substituent on C2 (in the C^4′^ position according to typical nucleoside labelling, see Scheme) and the hydroxyl substituent on C3 (C^3′^ position) are both axial or rather axial, whereas in mole 2 with 3′-endo puckering they are both equatorial. For the parent mol­ecule, d-deoxyadenosine, two crystal structures are available in the literature: one in pure form (Sato, 1984 ▸) and one as the monohydrate (Storr *et al.*, 2009 ▸). In the absence of water, the sugar adopts the 3′-endo confirmation with C^3′^ above the ^’^C^4′^–O–C^1′^ plane by 0.5 Å (Sato, 1984 ▸). In the presence of water, both the oxygen and hydrogen atoms of the hydroxyl substituent on C3′ are involved in hydrogen bonds with water and the sugar adopts the 3′-exo ring pucker with C^3′^ below the C^4′^–O–C^1′^ plane by 0.52 Å (Storr *et al.*, 2009 ▸). Hydrogen bonding in the crystal lattice apparently influences the ring pucker of deoxyribose moieties. In the present structure, bearing two mol­ecules with distinct ring pucker, the hydroxyl group bound to C3′ of the 3′-exo form (mole 1) is involved in one hydrogen bond as donor with water (O2—H2*O*⋯O7^ii^; see Table 1 ▸ for distances and angles) and that of the 3′-endo form (mole 2) is involved in bifurcated hydrogen bonding with two purine moieties (O5—H5*O*⋯N3^vi^ and N9—H9*N*⋯O5^ii^). Here C^3′^ is located 0.45 Å below (mole 1) and 0.82 Å above (mole 2) the respective C^4′^–O–C^1′^ planes. The methyl­ene–hydroxyl oxygen atom O4 on C19 (exo-C^4^’; mole 2) is involved as acceptor in a hydrogen bond with the water mol­ecule (O7—H70*O*⋯O4^vii^).

The aromatic six-membered rings of the two distinct mol­ecules are to some extent aligned with each other, forming pairs of phenyl and pyrimidine rings. They are neither coplanar nor perfectly overlaid, however. The respective planes for the mole 1 phenyl ring and mole 2 pyrimidine ring pair are at an angle of 15.1 (2)° and those of the mole 1 pyrimidine ring and mole 2 phenyl ring pair exhibit an angle of 14.6 (2)°. The centroid–centroid distance for the former pair is 3.652 (3) Å and it is 3.621 (3) Å for the latter. The intra­molecular angles between the planes of the aromatic six-membered ring systems are 36.8 (2) and 36.5 (2)° for mole 1 and mole 2, respectively, *i.e.* very similar.

In both mol­ecules, the conformation of the base with respect to the ribose moiety is *syn*, *i.e*. the pyrimidine ring and the deoxyribose moiety face the same direction. This is in contrast to the two known structures of d-deoxyadenosine (Sato, 1984 ▸; Storr *et al.*, 2009 ▸) and one structure of a derivative with an inverted configuration at C^3′^ (Robins *et al.*, 2007 ▸) but in accordance with the six other derivatives bearing a substit­uent at the C^8^ position (here C10 and C27) and no further substituents on deoxyadenosine that are reported in the literature (Vrábel *et al.*, 2007 ▸; Storr *et al.*, 2009 ▸, 2010 ▸). In all six cases, the substituents are aromatic in nature and more sterically demanding than the pyrimide ring of the purine base. The glycosidic torsion angles O—C^1′^—N^9^—C^4^ for the unsubstituted structures range from −94.76° (Robins *et al.*, 2007 ▸) to the more usual −178.74° (Storr *et al.*, 2009 ▸) for unsubstituted deoxyadenosine structures with an *anti* conformation. For substituted structures, which are in a *syn* conformation, these angles range from 48.73° for a *p*-fluoro-*p*-biphenyl substituent to 91.90° for a *p*-meth­oxy­phenyl substituent (Storr *et al.*, 2009 ▸). Here the respective torsion angles are 87.40° for mole 1 and 86.32° for mole 2, suggesting that this torsion angle and the pucker mode are independent of each other.

Bond lengths and angles of the purine bases in the two mol­ecules are very similar to previously reported values in related compounds. As is typical, the bond between C^5^ and C^6^ is the longest [mole 1, C8—C9, 1.409 (6) Å; mole 2, C25—C26, 1.393 (5) Å] and the bond between N^7^ and C^8^ is the shortest [mole 1, N5—C10, 1.315 (5) Å; mole 2, N10—C27, 1.323 (5) Å] of the planar heterocyclic ring system. For mole 2, these values are close to the low and high ends, respectively, of the reported values whereas those of mole 1 are rather average. For the deoxyribose ring, the shortest distance is usually found for the O—C^1′^ bond as is the case here. The locations of the longest bonds do vary. Most often it is the C^3′^—C^4′^ bond. However, in case of mole 1 it is C^2′^—C^3′^ [C3—C4, 1.532 (6) Å] and for mole 2 it is C^1′^—C^2′^ [C21—C22, 1.532 (5) Å], neither of which being unprecedented (Storr *et al.*, 2009 ▸, 2010 ▸). Most values found here fall inside the observed ranges for sugar moieties, the link between the sugar and base or the purine bases of the three known unsubstituted and the six substituted structures of d-2′-deoxyadenosine. The exceptions are C^3′^—C^4′^ for mole 1 [C2—C3, 1.504 (7) Å; range of literature known structures is 1.509–1.549 Å], C^4^—N^3^ for mole 2 [C23—N7, 1.332 (5) Å; range in the literature is 1.336–1.357 Å] and C^5^—N^7^ for both moles [C9—N5, 1.378 (5) Å, C26—N10, 1.377 (5) Å; range 1.380–1.394 Å], all of which being the shortest observed to date. No systematic influence of the substituent on the deoxyadenosine backbone with respect to distances and angles of the parent mol­ecule was observed as closely related compounds (phenyl-, *p*-meth­oxy­phenyl (both Storr *et al.*, 2009 ▸) and *p*-methyl­phenyl (this work) do not exhibit apparent similarities in this regard.

## Supra­molecular features   

**Figure 3:**
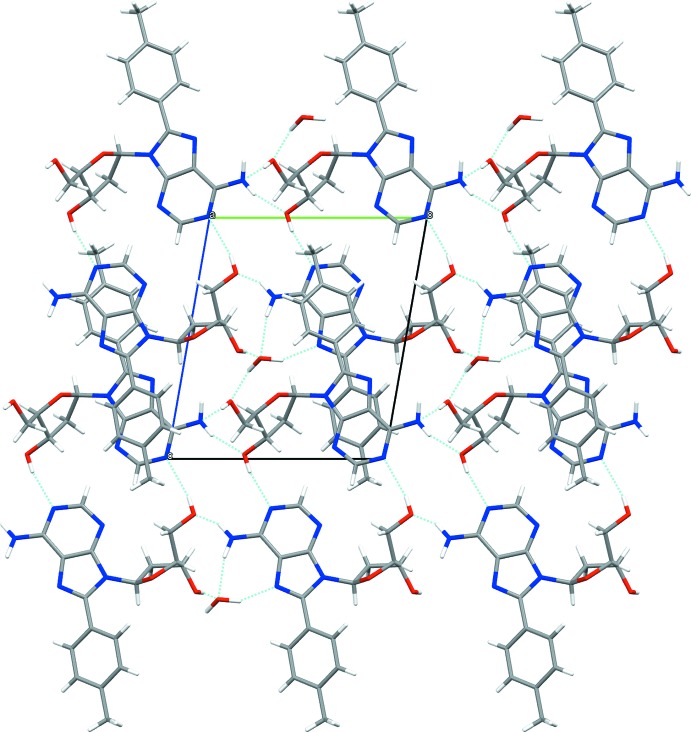
The crystal packing (*Mercury 3.19*; Macrae *et al.*, 2006 ▸) viewed along the *a* axis, showing the classical hydrogen bonds forming a three-dimensional network.

In the crystal, mol­ecules are linked by O—H⋯O, O—H⋯N, N—H⋯O, C—H⋯O and C—H⋯N classical and non-classical hydrogen-bonding contacts (Fig. 3 ▸ and Table 1 ▸), forming a three-dimensional network.

The water O atom (O7) utilizes both hydrogen atoms and both lone pairs to act as a hydrogen-bonding donor and acceptor with two nitro­gen atoms (N4—O7^iv^, O7—H7*O*⋯N5^vii^) of the purine base of mole 1, as donor to the hy­droxy­methyl oxygen atom (O7—H70*O*⋯O4^vii^) of mole 2 and as acceptor from the hydroxyl oxygen atom (O2*-*–H2*O*⋯O7^ii^) of mole 1. Mole 1 is directly linked to four neighbouring mol­ecules [(1) N4—H40*N*⋯O1^iii^, (2) N3⋯H5*O*—O5^i^, C7—H7⋯N7^i^, and O1—H1*O*⋯N8^i^, (3) O1⋯H40*N* ^ii^—N4, (4) C32—H32⋯O1^ii^] plus further four mediated by water. Mole 2 is in direct contact with six neighbouring mol­ecules [(1) C32—H32⋯O1^iii^, (2) O4—H4*O*⋯N9^v^, (3) N9—H9*N*⋯O5^ii^, (4) O5—H5*O*⋯N3^vi^, N7⋯H7—C7^vi^, and N8⋯H1*O*—O1^vi^,(5) O5⋯H9—N9^iii^, (6) N9⋯H4*O*—O4^viii^) plus further two mediated by water.

## Synthesis and crystallization   

The title compound, 8-(4-methyl­phen­yl)-d-2′-de­oxy­adenosine was synthesized based on a recently reported method (Bhilare *et al.*, 2016 ▸). The compound was obtained by the cross-coupling reaction of 8-bromo-2′-de­oxy­adenosine and 4-methyl­phenyl boronic acid in the presence of Pd(OAc)_2_ and PTABS (phospha-tris-aza-adamantyl-butane-saltone) ligand in neat water. The reaction was carried out in a Schlenk tube using a Schlenk system under a nitro­gen atmos­phere. All other reagents and solvents were purchased commercially and used without any further purification.

**Synthesis of 8-(4-methyl­phen­yl)-D-2′-de­oxy­adenosine:** To a solution of palladium acetate (1.12 mg, 1.0 mol %) and PTABS ligand (2.93 mg, 2.0 mol %) in degassed water (1.0 ml) at ambient temperature under N_2_ was added 8-bromo-d-2′-de­oxy­adenosine (0.5 mmol) and the solution was stirred for 5 min at 353 K. The reaction mixture was allowed to cool to room temperature and then 4-(meth­yl)phenyl boronic acid (0.75 mmol) was added along with tri­ethyl­amine (0.14 ml, 1 mmol) and degassed water (2.0 ml). The resulting solution was then stirred at 353 K for 12 h. The reaction progress was observed by TLC analysis. After the completion of the reaction, the solvent was removed *in vacuo* and the resultant residue obtained was purified using column chromatography in a CH_2_Cl_2_:MeOH solvent system (96:4) to afford the desired product as a white solid (143 mg, 84% yield). UV–visible absorption and fluorescence emission in methanol (10 µM) λ_abs_ = 301 nm, λ_fl_ = 371 nm. ^1^H NMR (400 MHz, DMSO-*d*_6_) δ 8.15 (*s*, 1H), 7.66 (*d*, *J* = 8.1 Hz, 2H), 7.47 (*d*, *J* = 8.2 Hz, 4H), 6.15 (*t*, *J* = 7.3 Hz, 1H), 5.56 (*s*, 1H), 5.30 (*d*, *J* = 18.0 Hz, 1H), 4.47 (*s*, 1H), 3.88 (*s*, 1H), 3.74–3.49 (*m*, 2H), 3.30 (*m*, 1H), 2.56 (*s*, 3H), 2.14 (*dd*, *J* = 12.2, 5.7 Hz, 1H). ^13^C NMR (100 MHz, DMSO-*d*_6_) δ 155.9, 151.7, 150.1, 149.7, 141.1, 129.6, 125.4, 125.2, 119.0, 88.1, 85.4, 71.2, 62.0, 45.0, 36.8. ESI–MS (*m*/*z*) = 374 (*M* ^+^ + H^+^). Analysis calculated for C_17_H_19_N_5_O_3_: C, 59.81; H, 5.61; N, 20.52. Found: C, 59.68; H, 5.46; N, 20.44.

## Refinement   

Crystal data, data collection and structure refinement details are summarized in Table 2 ▸.

The hydrogen atoms of water and the two –NH_2_ groups were located but refined with constraints (*SHELXL* instruction: SADI 0.05 O7 H7*O* O7 H70*O* and SADI 0.05 N4 H4*N* N4 H40*N* N9 H9*N* N9 H90*N*). The hydrogen atoms of the hydroxyl groups were attached with a riding model [*SHELXL* instruction: HFIX 147; *U*_iso_(H) = 1.5*U*_eq_(O)] with the orientation taken from the actually present electron density. When refined freely, the O—H distances became very long while using distance constraints did not improve the refinement compared with HFIX. The C-bound hydrogen atoms were included in calculated positions and treated as riding: C—H = 0.93–0.98 Å with *U*_iso_(H) = 1.5*U*_eq_(C) for methyl groups and *U*_iso_(H) = 1.2*U*_eq_(C) for all other C—H bonds.

## Supplementary Material

Crystal structure: contains datablock(s) I. DOI: 10.1107/S2056989017017212/zp2024sup1.cif

Structure factors: contains datablock(s) I. DOI: 10.1107/S2056989017017212/zp2024Isup2.hkl

CCDC reference: 1588387

Additional supporting information:  crystallographic information; 3D view; checkCIF report

## Figures and Tables

**Figure 2:**
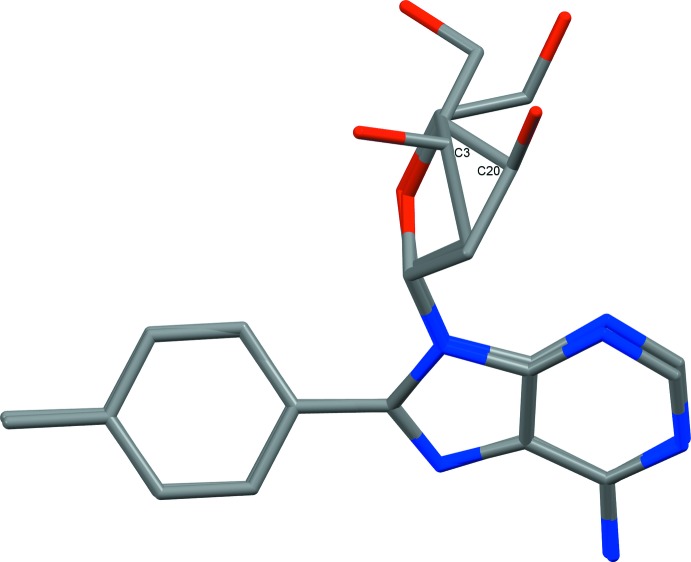
The two crystallographically independent mol­ecules of the asymmetric unit overlaid. The root-mean-square deviation (rmsd) and the maximum distance between atom positions are 0.8296 and 2.6760 Å, respectively.

**Table 1:** Hydrogen-bond geometry (Å, °)

*D*—H⋯*A*	*D*—H	H⋯*A*	*D*⋯*A*	*D*—H⋯*A*
O1—H1*O*⋯N8^i^	0.82	2.14	2.943 (5)	168
O2—H2*O*⋯O7^ii^	0.82	1.93	2.743 (5)	172
C1—H1*A*⋯N2	0.97	2.69	3.557 (7)	149
C4—H4*A*⋯N2	0.97	2.37	3.099 (5)	131
C16—H16⋯O3	0.93	2.49	3.257 (5)	140
C7—H7⋯N7^i^	0.93	2.66	3.374 (6)	135
N4—H40*N*⋯O1^iii^	0.91 (3)	2.27 (4)	3.033 (6)	142 (4)
N4—H4*N*⋯O7^iv^	0.92 (3)	2.05 (3)	2.955 (6)	166 (5)
O4—H4*O*⋯N9^v^	0.82	2.22	3.039 (5)	175
O5—H5*O*⋯N3^vi^	0.82	2.00	2.805 (4)	166
C20—H20⋯N7	0.98	2.56	3.201 (5)	123
C21—H21*B*⋯N7	0.97	2.47	2.992 (5)	113
C32—H32⋯O1^iii^	0.93	2.65	3.572 (6)	171
C33—H33⋯O6	0.93	2.50	3.288 (5)	143
N9—H9*N*⋯O5^ii^	0.92 (3)	2.01 (3)	2.903 (5)	164 (4)
O7—H7*O*⋯N5^vii^	0.98 (5)	1.87 (6)	2.789 (4)	154 (6)
O7—H70*O*⋯O4^vii^	0.99 (5)	1.82 (6)	2.761 (5)	159 (7)
O4—H4*O*⋯N9	0.82	2.22	3.039 (5)	175

Symmetry codes: (i) 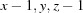; (ii) 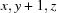; (iii) 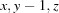; (iv) 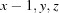; (v) 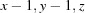; (vi) 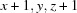; (vii) 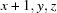.

**Table 2:** Experimental details

Crystal data	
Chemical formula	2C_17_H_19_N_5_O_3_·H_2_O
*M*_r_	700.76
Crystal system, space group	Triclinic, *P*1
Temperature (K)	293
*a*, *b*, *c* (Å)	7.3991 (15), 10.637 (2), 11.931 (2)
α, β, γ (°)	93.65 (3), 107.37 (3), 108.11 (3)
*V* (Å^3^)	839.1 (3)
*Z*	1
Radiation type	Mo *K*α
μ (mm^−1^)	0.10
Crystal size (mm)	0.29 × 0.17 × 0.12

Data collection	
Diffractometer	Stoe IPDS2T
No. of measured, independent and observed [*I* > 2σ(*I*)] reflections	7187, 5713, 4036
*R*_int_	0.033
(sin θ/λ)_max_ (Å^−1^)	0.638

Refinement	
*R*[*F* ^2^ > 2σ(*F* ^2^)], *wR*(*F* ^2^), *S*	0.041, 0.110, 1.00
No. of reflections	5713
No. of parameters	490
No. of restraints	10
H-atom treatment	H atoms treated by a mixture of independent and constrained refinement
Δρ_max_, Δρ_min_ (e Å^−3^)	0.29, −0.20
Absolute structure	Flack *x* determined using 1312 quotients [(*I* ^+^)−(*I* ^−^)]/[(*I* ^+^)+(*I* ^−^)] (Parsons et al., 2013 ▸)

Computer programs: *X-AREA* (Stoe & Cie, 2010 ▸), *SIR92* (Altomare *et al.*, 1994 ▸), *SHELXL2016* (Sheldrick, 2015 ▸) and *WinGX* (Farrugia, 2012 ▸), *XP* in *SHELXTL* (Sheldrick, 2008 ▸), *Mercury* (Macrae *et al.*, 2006 ▸) and *CIFTAB* (Sheldrick, 2015 ▸).

## supplementary crystallographic information

### Crystal data

**Table:**

2C_17_H_19_N_5_O_3_·H_2_O	*Z* = 1
*M**_r_* = 700.76	*F*(000) = 370
Triclinic, *P*1	*D*_x_ = 1.387 Mg m^−^^3^
*a* = 7.3991 (15) Å	Mo *K*α radiation, λ = 0.71073 Å
*b* = 10.637 (2) Å	Cell parameters from 7187 reflections
*c* = 11.931 (2) Å	θ = 6.4–53.9°
α = 93.65 (3)°	µ = 0.10 mm^−^^1^
β = 107.37 (3)°	*T* = 293 K
γ = 108.11 (3)°	Rhomb, colourless
*V* = 839.1 (3) Å^3^	0.29 × 0.17 × 0.12 mm

### Data collection

**Table:**

Stoe IPDS2T diffractometer	4036 reflections with *I* > 2σ(*I*)
Radiation source: fine-focus sealed tube	*R*_int_ = 0.033
Detector resolution: 6.67 pixels mm^-1^	θ_max_ = 27.0°, θ_min_ = 3.2°
ω scans	*h* = −9→9
7187 measured reflections	*k* = −13→13
5713 independent reflections	*l* = −15→14

### Refinement

**Table:**

Refinement on *F*^2^	Hydrogen site location: mixed
Least-squares matrix: full	H atoms treated by a mixture of independent and constrained refinement
*R*[*F*^2^ > 2σ(*F*^2^)] = 0.041	*w* = 1/[σ^2^(*F*_o_^2^) + (0.065*P*)^2^] where *P* = (*F*_o_^2^ + 2*F*_c_^2^)/3
*wR*(*F*^2^) = 0.110	(Δ/σ)_max_ < 0.001
*S* = 1.00	Δρ_max_ = 0.28 e Å^−^^3^
5713 reflections	Δρ_min_ = −0.20 e Å^−^^3^
490 parameters	Absolute structure: Flack *x* determined using 1312 quotients [(*I*^+^)-(*I*^-^)]/[(*I*^+^)+(*I*^-^)] (Parsons et al., 2013)
10 restraints	

### Special details

**Table:**

Geometry. All esds (except the esd in the dihedral angle between two l.s. planes) are estimated using the full covariance matrix. The cell esds are taken into account individually in the estimation of esds in distances, angles and torsion angles; correlations between esds in cell parameters are only used when they are defined by crystal symmetry. An approximate (isotropic) treatment of cell esds is used for estimating esds involving l.s. planes.

### Fractional atomic coordinates and isotropic or equivalent isotropic displacement parameters (Å^2^)

**Table:**

	*x*	*y*	*z*	*U*_iso_*/*U*_eq_	
O1	−0.0436 (7)	1.1671 (4)	0.2298 (3)	0.0884 (13)	
H1O	−0.097552	1.129571	0.160023	0.133*	
O2	0.4464 (5)	1.2386 (3)	0.5518 (3)	0.0652 (9)	
H2O	0.563957	1.269834	0.554191	0.098*	
O3	0.0583 (4)	1.0235 (3)	0.4948 (3)	0.0600 (8)	
N1	0.0975 (5)	0.8132 (3)	0.4909 (3)	0.0420 (8)	
N2	0.0200 (5)	0.7555 (3)	0.2751 (3)	0.0502 (9)	
N3	−0.1330 (5)	0.5172 (3)	0.2056 (3)	0.0519 (9)	
N4	−0.2060 (6)	0.3617 (4)	0.3286 (4)	0.0615 (11)	
N5	−0.0280 (5)	0.6057 (3)	0.5288 (3)	0.0433 (8)	
C1	−0.0454 (10)	1.0721 (5)	0.3029 (5)	0.0878 (19)	
H1A	−0.018480	0.998584	0.266890	0.105*	
H1B	−0.178735	1.036543	0.309416	0.105*	
C2	0.1090 (7)	1.1277 (4)	0.4265 (4)	0.0565 (12)	
H2	0.095003	1.209404	0.459880	0.068*	
C3	0.3265 (7)	1.1525 (4)	0.4398 (4)	0.0525 (11)	
H3	0.361711	1.190427	0.373226	0.063*	
C4	0.3418 (6)	1.0125 (4)	0.4446 (4)	0.0466 (9)	
H4A	0.293488	0.959503	0.365506	0.056*	
H4B	0.478870	1.017134	0.485890	0.056*	
C5	0.2028 (6)	0.9575 (3)	0.5149 (4)	0.0409 (8)	
H5	0.284651	0.982718	0.599710	0.049*	
C6	0.0214 (5)	0.7262 (4)	0.3837 (3)	0.0418 (9)	
C7	−0.0600 (7)	0.6446 (4)	0.1936 (4)	0.0528 (11)	
H7	−0.065826	0.658151	0.116535	0.063*	
C8	−0.1309 (6)	0.4903 (4)	0.3151 (4)	0.0476 (10)	
C9	−0.0530 (6)	0.6002 (4)	0.4092 (4)	0.0429 (9)	
C10	0.0600 (5)	0.7341 (4)	0.5750 (3)	0.0399 (9)	
C11	0.1178 (5)	0.7870 (4)	0.7025 (4)	0.0405 (9)	
C12	0.1870 (6)	0.7128 (4)	0.7855 (4)	0.0475 (10)	
H12	0.194438	0.630720	0.759860	0.057*	
C13	0.2450 (6)	0.7593 (5)	0.9061 (4)	0.0547 (11)	
H13	0.291305	0.707810	0.960356	0.066*	
C14	0.2358 (6)	0.8806 (5)	0.9481 (4)	0.0547 (11)	
C15	0.1588 (7)	0.9530 (5)	0.8648 (4)	0.0544 (11)	
H15	0.147807	1.033659	0.890854	0.065*	
C16	0.0983 (6)	0.9064 (4)	0.7434 (4)	0.0497 (10)	
H16	0.044545	0.955128	0.688939	0.060*	
C17	0.3074 (9)	0.9341 (7)	1.0797 (5)	0.0809 (16)	
H17A	0.441281	0.999192	1.103230	0.121*	
H17B	0.308729	0.861573	1.123501	0.121*	
H17C	0.217986	0.975718	1.096240	0.121*	
H40N	−0.216 (7)	0.295 (4)	0.273 (4)	0.068 (15)*	
H4N	−0.200 (9)	0.342 (6)	0.404 (3)	0.085 (18)*	
O4	0.0629 (5)	0.2449 (4)	0.7618 (3)	0.0816 (12)	
H4O	−0.028481	0.212598	0.788312	0.122*	
O5	0.6871 (5)	0.3508 (3)	0.9814 (3)	0.0555 (8)	
H5O	0.744943	0.388280	1.051057	0.083*	
O6	0.4002 (4)	0.4607 (2)	0.7339 (2)	0.0446 (6)	
N6	0.6124 (5)	0.6764 (3)	0.7384 (3)	0.0388 (7)	
N7	0.7163 (5)	0.7581 (3)	0.9512 (3)	0.0472 (8)	
N8	0.7603 (5)	0.9933 (3)	0.9942 (3)	0.0491 (9)	
N9	0.7062 (6)	1.1223 (3)	0.8448 (3)	0.0503 (9)	
N10	0.5980 (5)	0.8685 (3)	0.6717 (3)	0.0409 (7)	
C18	0.2362 (7)	0.3301 (5)	0.8554 (5)	0.0633 (13)	
H18A	0.261470	0.284895	0.923486	0.076*	
H18B	0.213785	0.411095	0.879997	0.076*	
C19	0.4151 (6)	0.3660 (4)	0.8140 (3)	0.0432 (9)	
H19	0.421093	0.285073	0.773577	0.052*	
C20	0.6147 (6)	0.4399 (4)	0.9122 (3)	0.0410 (9)	
H20	0.599021	0.508260	0.963841	0.049*	
C21	0.7459 (6)	0.5074 (4)	0.8419 (3)	0.0435 (9)	
H21A	0.808272	0.448407	0.816158	0.052*	
H21B	0.850804	0.589849	0.889000	0.052*	
C22	0.5983 (6)	0.5365 (3)	0.7348 (3)	0.0392 (8)	
H22	0.616428	0.504592	0.661376	0.047*	
C23	0.6656 (5)	0.7723 (4)	0.8371 (3)	0.0395 (9)	
C24	0.7619 (7)	0.8731 (4)	1.0220 (4)	0.0544 (11)	
H24	0.800784	0.869971	1.103164	0.065*	
C25	0.7055 (5)	1.0019 (4)	0.8780 (4)	0.0415 (9)	
C26	0.6558 (6)	0.8884 (4)	0.7942 (3)	0.0391 (8)	
C27	0.5714 (5)	0.7408 (4)	0.6401 (3)	0.0384 (9)	
C28	0.5122 (5)	0.6778 (4)	0.5165 (3)	0.0397 (9)	
C29	0.5855 (6)	0.7548 (4)	0.4390 (4)	0.0466 (10)	
H29	0.671644	0.843321	0.467040	0.056*	
C30	0.5305 (7)	0.6997 (5)	0.3202 (4)	0.0537 (11)	
H30	0.580047	0.752242	0.269235	0.064*	
C31	0.4041 (7)	0.5690 (5)	0.2762 (4)	0.0534 (11)	
C32	0.3301 (6)	0.4924 (4)	0.3528 (4)	0.0514 (10)	
H32	0.244670	0.403800	0.324119	0.062*	
C33	0.3815 (6)	0.5460 (4)	0.4717 (4)	0.0455 (9)	
H33	0.328588	0.493749	0.521693	0.055*	
C34	0.3460 (9)	0.5077 (6)	0.1468 (4)	0.0801 (16)	
H34A	0.390460	0.432367	0.142542	0.120*	
H34B	0.408834	0.573573	0.105929	0.120*	
H34C	0.202055	0.478320	0.110063	0.120*	
H9N	0.703 (7)	1.185 (4)	0.900 (3)	0.056 (13)*	
H90N	0.621 (8)	1.113 (7)	0.768 (3)	0.10 (2)*	
O7	0.8496 (5)	0.3480 (3)	0.5826 (3)	0.0684 (9)	
H7O	0.909 (10)	0.446 (5)	0.590 (6)	0.11 (2)*	
H70O	0.937 (11)	0.305 (7)	0.632 (7)	0.14 (3)*	

### Atomic displacement parameters (Å^2^)

**Table:**

	*U*^11^	*U*^22^	*U*^33^	*U*^12^	*U*^13^	*U*^23^
O1	0.142 (4)	0.065 (2)	0.051 (2)	0.051 (2)	0.006 (2)	0.0055 (17)
O2	0.067 (2)	0.0471 (17)	0.061 (2)	−0.0004 (15)	0.0191 (15)	−0.0151 (14)
O3	0.0551 (17)	0.0440 (15)	0.086 (2)	0.0229 (13)	0.0232 (16)	0.0184 (15)
N1	0.0483 (18)	0.0340 (16)	0.0365 (18)	0.0116 (13)	0.0072 (14)	0.0055 (13)
N2	0.066 (2)	0.0410 (18)	0.0357 (19)	0.0174 (16)	0.0061 (15)	0.0073 (15)
N3	0.066 (2)	0.0388 (18)	0.039 (2)	0.0180 (16)	0.0023 (16)	0.0025 (15)
N4	0.085 (3)	0.0343 (18)	0.055 (3)	0.0153 (17)	0.015 (2)	0.0023 (17)
N5	0.0462 (17)	0.0356 (17)	0.043 (2)	0.0120 (14)	0.0109 (14)	0.0056 (14)
C1	0.112 (5)	0.058 (3)	0.072 (4)	0.038 (3)	−0.007 (3)	0.002 (3)
C2	0.072 (3)	0.039 (2)	0.053 (3)	0.025 (2)	0.008 (2)	0.0082 (19)
C3	0.074 (3)	0.034 (2)	0.039 (2)	0.0078 (19)	0.018 (2)	0.0034 (17)
C4	0.052 (2)	0.040 (2)	0.045 (2)	0.0105 (17)	0.0177 (17)	0.0030 (17)
C5	0.0447 (19)	0.0314 (17)	0.044 (2)	0.0133 (15)	0.0111 (16)	0.0040 (15)
C6	0.045 (2)	0.0347 (19)	0.040 (2)	0.0141 (16)	0.0064 (17)	0.0046 (17)
C7	0.067 (3)	0.049 (2)	0.035 (2)	0.021 (2)	0.0065 (19)	0.0053 (19)
C8	0.050 (2)	0.038 (2)	0.048 (3)	0.0173 (17)	0.0063 (18)	0.0025 (18)
C9	0.045 (2)	0.039 (2)	0.039 (2)	0.0165 (16)	0.0044 (16)	0.0043 (16)
C10	0.0394 (19)	0.040 (2)	0.039 (2)	0.0143 (16)	0.0095 (16)	0.0091 (17)
C11	0.0387 (19)	0.0397 (19)	0.040 (2)	0.0111 (16)	0.0116 (16)	0.0051 (16)
C12	0.048 (2)	0.043 (2)	0.049 (3)	0.0150 (17)	0.0143 (18)	0.0099 (18)
C13	0.054 (2)	0.062 (3)	0.048 (3)	0.018 (2)	0.018 (2)	0.020 (2)
C14	0.050 (2)	0.063 (3)	0.042 (2)	0.008 (2)	0.0156 (19)	0.005 (2)
C15	0.059 (3)	0.051 (2)	0.052 (3)	0.0171 (19)	0.020 (2)	0.002 (2)
C16	0.055 (2)	0.051 (2)	0.042 (2)	0.0219 (19)	0.0118 (18)	0.0064 (18)
C17	0.081 (3)	0.102 (4)	0.047 (3)	0.023 (3)	0.016 (2)	0.001 (3)
O4	0.0562 (19)	0.068 (2)	0.093 (3)	−0.0004 (17)	0.0064 (18)	0.029 (2)
O5	0.086 (2)	0.0397 (15)	0.0366 (16)	0.0298 (14)	0.0064 (14)	0.0063 (12)
O6	0.0470 (14)	0.0376 (13)	0.0424 (15)	0.0119 (11)	0.0075 (12)	0.0120 (12)
N6	0.0492 (17)	0.0314 (15)	0.0333 (17)	0.0138 (13)	0.0108 (13)	0.0039 (13)
N7	0.065 (2)	0.0362 (17)	0.0339 (18)	0.0168 (15)	0.0098 (15)	0.0020 (14)
N8	0.060 (2)	0.0368 (18)	0.044 (2)	0.0155 (15)	0.0099 (16)	0.0006 (15)
N9	0.065 (2)	0.0350 (18)	0.049 (2)	0.0177 (16)	0.0172 (18)	0.0046 (16)
N10	0.0448 (17)	0.0340 (16)	0.0399 (19)	0.0129 (13)	0.0098 (14)	0.0051 (13)
C18	0.058 (3)	0.054 (3)	0.066 (3)	0.009 (2)	0.015 (2)	0.020 (2)
C19	0.058 (2)	0.0299 (17)	0.037 (2)	0.0134 (16)	0.0098 (17)	0.0085 (15)
C20	0.055 (2)	0.0338 (18)	0.034 (2)	0.0196 (16)	0.0113 (17)	0.0053 (15)
C21	0.047 (2)	0.042 (2)	0.043 (2)	0.0215 (16)	0.0103 (17)	0.0077 (17)
C22	0.050 (2)	0.0331 (19)	0.036 (2)	0.0155 (16)	0.0153 (16)	0.0077 (15)
C23	0.043 (2)	0.0366 (19)	0.038 (2)	0.0147 (16)	0.0115 (16)	0.0057 (17)
C24	0.075 (3)	0.043 (2)	0.039 (2)	0.021 (2)	0.011 (2)	0.0041 (18)
C25	0.041 (2)	0.0349 (19)	0.047 (2)	0.0126 (15)	0.0130 (17)	0.0060 (17)
C26	0.0433 (19)	0.0321 (18)	0.040 (2)	0.0118 (15)	0.0125 (16)	0.0043 (15)
C27	0.042 (2)	0.038 (2)	0.035 (2)	0.0134 (16)	0.0122 (16)	0.0085 (16)
C28	0.044 (2)	0.0384 (19)	0.037 (2)	0.0178 (16)	0.0091 (16)	0.0068 (16)
C29	0.051 (2)	0.045 (2)	0.046 (2)	0.0167 (17)	0.0183 (18)	0.0121 (18)
C30	0.062 (3)	0.064 (3)	0.042 (3)	0.023 (2)	0.023 (2)	0.017 (2)
C31	0.062 (3)	0.066 (3)	0.038 (2)	0.033 (2)	0.014 (2)	0.008 (2)
C32	0.058 (2)	0.046 (2)	0.041 (2)	0.0157 (19)	0.0072 (19)	−0.0026 (18)
C33	0.051 (2)	0.044 (2)	0.041 (2)	0.0163 (17)	0.0142 (17)	0.0069 (17)
C34	0.104 (4)	0.103 (4)	0.036 (3)	0.047 (4)	0.018 (3)	0.000 (3)
O7	0.068 (2)	0.0545 (19)	0.066 (2)	0.0063 (16)	0.0117 (16)	0.0164 (17)

### Geometric parameters (Å, º)

**Table:**

O1—C1	1.375 (7)	O4—C18	1.417 (6)
O2—C3	1.429 (5)	O5—C20	1.421 (4)
O3—C5	1.424 (5)	O6—C22	1.433 (5)
O3—C2	1.440 (5)	O6—C19	1.438 (4)
N1—C6	1.381 (5)	N6—C23	1.379 (5)
N1—C10	1.385 (5)	N6—C27	1.400 (5)
N1—C5	1.454 (4)	N6—C22	1.456 (5)
N2—C7	1.328 (5)	N7—C24	1.329 (5)
N2—C6	1.350 (5)	N7—C23	1.332 (5)
N3—C7	1.329 (6)	N8—C25	1.341 (5)
N3—C8	1.352 (6)	N8—C24	1.344 (5)
N4—C8	1.347 (6)	N9—C25	1.364 (5)
N5—C10	1.315 (5)	N10—C27	1.323 (5)
N5—C9	1.378 (5)	N10—C26	1.377 (5)
C1—C2	1.515 (7)	C18—C19	1.497 (7)
C2—C3	1.504 (7)	C19—C20	1.517 (5)
C3—C4	1.532 (6)	C20—C21	1.513 (6)
C4—C5	1.519 (6)	C21—C22	1.532 (5)
C6—C9	1.373 (5)	C23—C26	1.382 (5)
C8—C9	1.409 (6)	C25—C26	1.393 (5)
C10—C11	1.472 (6)	C27—C28	1.456 (6)
C11—C12	1.384 (6)	C28—C33	1.392 (5)
C11—C16	1.396 (6)	C28—C29	1.393 (6)
C12—C13	1.381 (6)	C29—C30	1.385 (6)
C13—C14	1.384 (7)	C30—C31	1.374 (6)
C14—C15	1.391 (7)	C31—C32	1.384 (6)
C14—C17	1.507 (7)	C31—C34	1.515 (7)
C15—C16	1.389 (6)	C32—C33	1.386 (6)
			
C5—O3—C2	109.4 (3)	C22—O6—C19	109.1 (3)
C6—N1—C10	105.9 (3)	C23—N6—C27	105.6 (3)
C6—N1—C5	128.4 (3)	C23—N6—C22	128.1 (3)
C10—N1—C5	125.7 (3)	C27—N6—C22	126.3 (3)
C7—N2—C6	111.0 (3)	C24—N7—C23	111.0 (3)
C7—N3—C8	118.3 (4)	C25—N8—C24	116.8 (3)
C10—N5—C9	104.8 (3)	C27—N10—C26	104.8 (3)
O1—C1—C2	112.7 (4)	O4—C18—C19	109.7 (4)
O3—C2—C3	104.9 (3)	O6—C19—C18	109.7 (3)
O3—C2—C1	104.4 (4)	O6—C19—C20	103.1 (3)
C3—C2—C1	117.3 (5)	C18—C19—C20	114.2 (3)
O2—C3—C2	107.1 (4)	O5—C20—C21	114.2 (3)
O2—C3—C4	109.9 (4)	O5—C20—C19	111.7 (3)
C2—C3—C4	103.0 (3)	C21—C20—C19	101.8 (3)
C5—C4—C3	100.3 (3)	C20—C21—C22	103.9 (3)
O3—C5—N1	109.0 (3)	O6—C22—N6	107.4 (3)
O3—C5—C4	107.9 (3)	O6—C22—C21	106.0 (3)
N1—C5—C4	117.1 (3)	N6—C22—C21	115.5 (3)
N2—C6—C9	125.9 (4)	N7—C23—N6	127.9 (3)
N2—C6—N1	128.3 (3)	N7—C23—C26	126.1 (3)
C9—C6—N1	105.8 (3)	N6—C23—C26	106.1 (3)
N2—C7—N3	129.7 (4)	N7—C24—N8	129.8 (4)
N4—C8—N3	118.9 (4)	N8—C25—N9	119.3 (4)
N4—C8—C9	123.5 (4)	N8—C25—C26	119.1 (3)
N3—C8—C9	117.5 (4)	N9—C25—C26	121.6 (4)
C6—C9—N5	111.0 (3)	N10—C26—C23	111.2 (3)
C6—C9—C8	117.6 (4)	N10—C26—C25	131.7 (3)
N5—C9—C8	131.3 (4)	C23—C26—C25	117.1 (3)
N5—C10—N1	112.4 (3)	N10—C27—N6	112.3 (3)
N5—C10—C11	123.6 (3)	N10—C27—C28	122.9 (3)
N1—C10—C11	123.9 (3)	N6—C27—C28	124.7 (3)
C12—C11—C16	118.4 (4)	C33—C28—C29	118.7 (4)
C12—C11—C10	118.7 (4)	C33—C28—C27	123.2 (3)
C16—C11—C10	122.8 (4)	C29—C28—C27	118.1 (3)
C13—C12—C11	120.7 (4)	C30—C29—C28	120.2 (4)
C12—C13—C14	121.5 (4)	C31—C30—C29	121.2 (4)
C13—C14—C15	117.9 (4)	C30—C31—C32	118.8 (4)
C13—C14—C17	121.5 (5)	C30—C31—C34	121.5 (4)
C15—C14—C17	120.6 (5)	C32—C31—C34	119.8 (4)
C16—C15—C14	121.0 (4)	C31—C32—C33	120.9 (4)
C15—C16—C11	120.4 (4)	C32—C33—C28	120.2 (4)

### Hydrogen-bond geometry (Å, º)

**Table:**

*D*—H···*A*	*D*—H	H···*A*	*D*···*A*	*D*—H···*A*
O1—H1*O*···N8^i^	0.82	2.14	2.943 (5)	168
O2—H2*O*···O7^ii^	0.82	1.93	2.743 (5)	172
C1—H1*A*···N2	0.97	2.69	3.557 (7)	149
C4—H4*A*···N2	0.97	2.37	3.099 (5)	131
C16—H16···O3	0.93	2.49	3.257 (5)	140
C7—H7···N7^i^	0.93	2.66	3.374 (6)	135
N4—H40*N*···O1^iii^	0.91 (3)	2.27 (4)	3.033 (6)	142 (4)
N4—H4*N*···O7^iv^	0.92 (3)	2.05 (3)	2.955 (6)	166 (5)
O4—H4*O*···N9^v^	0.82	2.22	3.039 (5)	175
O5—H5*O*···N3^vi^	0.82	2.00	2.805 (4)	166
C20—H20···N7	0.98	2.56	3.201 (5)	123
C21—H21*B*···N7	0.97	2.47	2.992 (5)	113
C32—H32···O1^iii^	0.93	2.65	3.572 (6)	171
C33—H33···O6	0.93	2.50	3.288 (5)	143
N9—H9*N*···O5^ii^	0.92 (3)	2.01 (3)	2.903 (5)	164 (4)
O7—H7*O*···N5^vii^	0.98 (5)	1.87 (6)	2.789 (4)	154 (6)
O7—H70*O*···O4^vii^	0.99 (5)	1.82 (6)	2.761 (5)	159 (7)
O4—H4*O*···N9	0.82	2.22	3.039 (5)	175

Symmetry codes: (i) *x*−1, *y*, *z*−1; (ii) *x*, *y*+1, *z*; (iii) *x*, *y*−1, *z*; (iv) *x*−1, *y*, *z*; (v) *x*−1, *y*−1, *z*; (vi) *x*+1, *y*, *z*+1; (vii) *x*+1, *y*, *z*.
